# Fused Filament Fabrication Process: A Review of Numerical Simulation Techniques

**DOI:** 10.3390/polym13203534

**Published:** 2021-10-14

**Authors:** Ans Al Rashid, Muammer Koç

**Affiliations:** 1 Division of Sustainable Development, College of Science and Engineering, Hamad Bin Khalifa University, Qatar Foundation, Doha 34110, Qatar; mkoc@hbku.edu.qa; 2 Faculty of Engineering, University of Karabük, Karabük 78050, Turkey

**Keywords:** additive manufacturing, 3D printing, computational modeling, simulation technique, computational fluid dynamics

## Abstract

Three-dimensional printing (3DP), also known as additive manufacturing (AM), has rapidly evolved over the past few decades. Researchers around the globe have been putting their efforts into AM processes improvement and materials development. One of the most widely used extrusion-based technology under AM processes is Fused Deposition Modeling (FDM), also known as Fused Filament Fabrication (FFF). Numerical simulation tools are being employed to predict the FFF process complexities and material behavior. These tools allow exploring candidate materials for their potential use in the FFF process and process improvements. The prime objective of this study is to provide a comprehensive review of state-of-the-art scientific achievements in numerical simulations of the FFF process for polymers and their composites. The first section presents an in-depth discussion of the FFF process’s physical phenomena and highlights the multi-level complexity. The subsequent section discusses the research efforts, specifically on numerical simulation techniques reported in the literature for simulation of the FFF process. Finally, conclusions are drawn based on the reviewed literature, and future research directions are identified.

## 1. Introduction

Three-dimensional printing, also known as additive manufacturing (AM), has rapidly evolved over the past few decades [1,2]. AM processes allow the fabrication of three-dimensional and functional components through successive additions of 2D layers [3]. AM was first introduced by Hull [3], since then, researchers have established several process technologies and novel materials [4]. These processes have attracted the research interest due to higher flexibility in the design and the manufacturing of highly customizable parts, rapid prototyping of conceptual products, waste reduction, lower risk of human error, and higher precision and accuracy than conventional manufacturing processes [5,6,7]. AM processes are now being widely adopted in several industrial sectors owing to these advantages [8,9].

Fused Deposition Modeling (FDM), also known as Fused Filament Fabrication (FFF), one of the most widely used AM processes, was proposed and developed by Scott Crump at Stratasys [10]. In the FFF process, the material is supplied to the 3D printer in the form of filaments. The extruder in any FFF-based 3D printer generally contains a feed material control mechanism, heating chamber, and nozzle. The material in the filaments form is fed to the control system, which controls the feed rate. Then the filament moves through the heating chamber, which converts it into a semi-solid phase and then passes through the nozzle for deposition on the printing bed [11]. The mechanical and thermal controls in the extruder govern the overall printing process and depend upon the feed-stock material properties. Thermoplastics, particles reinforced polymers, and hydrogels have been fabricated using the FFF process [12].

Researchers around the globe have been putting their efforts into AM processes improvement and novel materials over the past few decades [13,14]. The research efforts include process printing speed, printer build volume, and production rate [15]. The materials portfolio has also included short and continuous fiber-reinforced polymer composites for the FFF process [16,17,18]. Aside from the extensive experimental investigations on the FFF process reported in the literature, the numerical tools employed are still limited.

The prime objective of this study is to provide a comprehensive review of the state-of-the-art scientific achievements in numerical simulations of the FFF process for polymers and their composites. The first section presents an in-depth discussion of the FFF process’s physical phenomena and highlights the multi-level complexity. The subsequent section discusses the research efforts, specifically on numerical simulation methods reported in the literature for simulation of the FFF process. Finally, conclusions are drawn based on the reviewed literature, and future research directions are identified.

## 2. Physics Involved in Fused Filament Fabrication Process

**Figure 1:**
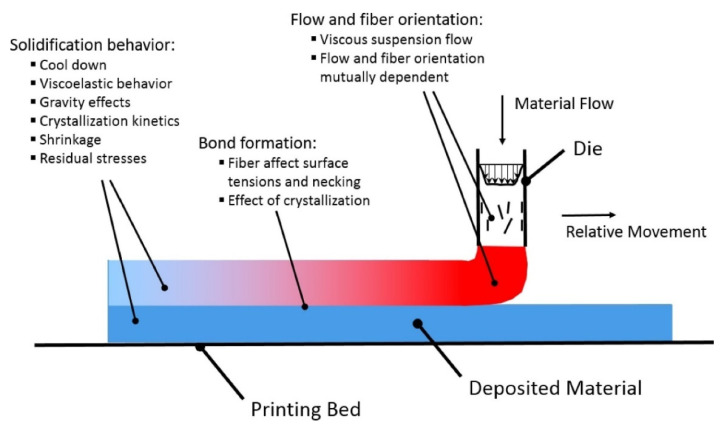
Schematic diagram of the FFF process. Reprinted with permission from reference [20]. Copyright 2018, Elsevier.

This section presents the physical phenomena involved in the FFF process. An in-depth understanding of this phenomenon is essential to improve the process and 3D printed part quality. The overall FFF process can be divided into three phases; material flow through the nozzle and deposition on the print bed or already deposited material (extrusion), interaction of deposited beads to make a bond (fusion), and the cooling process (solidification), as elaborated in Figure 1.

The first process involves material flow through the nozzle body, nozzle orifice, and deposition [19]. This phase is of great interest, especially in the 3DP of fiber-reinforced polymer composites, as fiber orientation results from material flow through the orifice and regulates mechanical behavior (anisotropy) of the fabricated part. The flow behavior and fiber orientation are interdependent, as a viscous fiber-suspension flow is obtained for materials with considerable fiber volume fractions. Shear alignment phenomena and converging zone in nozzle determine the fiber orientation in printed beads. The viscosity of the material is also dependent upon fiber orientation. For example, the extensional viscosity parallel to fiber alignment is multiple folds higher than in the transverse direction.

In the second phase, the deposited material reheats or re-melts the already deposited material beads [21]. The bonding between the extruded filaments is highly dependent on this wetting phenomenon [22]. The wetting phenomena regulate the contact area between the deposited filaments, as prolonged interface exposure at elevated temperatures assists the merging of adjacent beads and polymer chains diffusion [23]. The viscosity of beads in the transverse direction and surface tension of the material controls this wetting process. A limited temperature range is available for perfect bead formation between the layers, as viscosity is temperature-dependent [24]. Diffusion-based fusion is observed between the beads at higher temperatures after an interface has been formed. However, the diffusion process is obstructed due to reduced molecular mobility at lower temperatures. Therefore, a critical factor for adequate bonding is the temperature history of printed material. Additional complexity arises for semi-crystalline materials, as the viscosity of such materials rapidly increases at crystallization temperature, interrupting the bond formation process [25].

Lastly, the deposited material begins to solidify as it cools down. The cooling process during and post-printing is governed by convective and radiative heat losses on external surfaces of the beads and conductive heat transfer at beads contact points and printing bed. If the material is deposited at a higher speed, i.e., a large amount of material is deposited in a shorter time, it will not allow the pre-laid layer to cool down sufficiently before depositing the next layer [26]. It will result in sagging due to gravity and print failure. The material converts to a viscoelastic solid from a viscous fluid during the cooling process and starts shrinking depending upon the coefficient of thermal expansion (CTE). The internal stresses begin to develop due to material stiffness produced by the solidification process and restriction due to bead fusion. Viscoelastic relaxation and material deformations assist a fraction of internal stresses to be released. However, for semi-crystalline polymers, the mechanical and thermal properties change during the crystallization process. For such materials, additional strains are observed that further cause internal stresses and part deformations, resulting in altered mechanical properties [27]. The presence of fibers aligned in the printing direction also affects the thermal properties due to increased thermal conductivity. Thermomechanical and crystallization effects constraints the shrinkage process in the bead direction, but not to the same degree in the lateral direction [28]. The physical phenomena involved in the FFF process highlight its complexity. Therefore, the above-discussed process physics and interactions must be considered for more realistic process simulation and modeling.

## 3. Numerical Simulation Techniques

There has been an increasing interest in modeling and simulation of the FFF process since the commercial availability of 3D printers. The past and ongoing research related to computational modeling of the FFF process can be broken down into two domains. In the first domain, thermal variations and fluid flow behavior of the material in the heating zone are modeled. Overall melt flow behavior of the material inside the printer extruder depends upon the heat capacities of the nozzle and liquefier and the thermal properties of the material. Secondly, when the material is extruded through the nozzle, sudden pressure change causes swelling following the deposition. This stage is of great interest as material flow outside the nozzle, bead shape, bonding of beads, and residual stresses are governed by these factors. Following, we present the computational works related to the FFF of polymers and polymer composites, also reported in Table 1.

### 3.1. Melt Flow Behavior

The first study on modeling and characterization of material flow inside the liquefier and nozzle in the FFF process was conducted by Bellini [54]. The material flow through the FFF extrusion system (i.e., liquefier, nozzle, and die) for ceramic materials were modeled and discussed for PZT/ECG9 material. The pertinent achievement of this study included a tool for the controlled deposition process. A model was developed for the selection of the optimal nozzle shape from extensive experimental results. The velocity and temperature fields of the material during the deposition process were simulated and compared with experimental results. Moreover, the incorporated particles were observed to be aligned in the printing direction of extruded strands, but this effect was not included in simulations.

Although the first study in this area considered ceramic materials, it laid the foundation for the numerical analysis of 3D printed polymers. Ramanath et al. [29] numerically investigated the melt flow behavior of Poly-ε-caprolactone (PCL) biomaterial processed via the FFF process. PCL is an emerging material in the biomedical field, as it has found its applications in this sector due to its biodegradability [55]. An accurate channel model was employed to study the velocity gradient, pressure variations, and thermal behavior by varying filament velocity at entry and nozzle shape and the angle at the exit. From mathematical and numerical results, velocity profiles and pressure gradients strongly depended on the flow channel parameters. Temperature profiles revealed that material completely liquefied within 35% of the channel length, which is an important outcome and can be implemented to predict the melt flow behavior of other materials.

Researchers have also considered ABS material for numerical studies. ABS material can is widely regarded for the synthesis of polymer composites [56]. This material has found its mechanical applications due to its impressive mechanical and physical properties and chemical resistance [57,58]. Monzón et al. [31] conducted a theoretical and experimental study to evaluate the potential of using a 0.05 mm diameter nozzle for the FFF process. The material swelling and filament cooling during the deposition process was studied for ABS. A conventional FFF printer was used to extrapolate the information for micro-additive fused deposition (MAFD). Die swelling was attributed to nozzle and envelope temperature, where nozzle temperature plays a more significant role. In addition, due to the location of the heating element, temperature variations were observed along the nozzle length with the lower temperature at the nozzle exit. The nozzle diameter and extrudate diameter ratio were used for the proposed extrapolation and termed the swelling diameter factor. It was concluded that the flow volume could be reduced multiple folds using fine nozzle diameters (i.e., up to 215 times).

**Figure 2:**
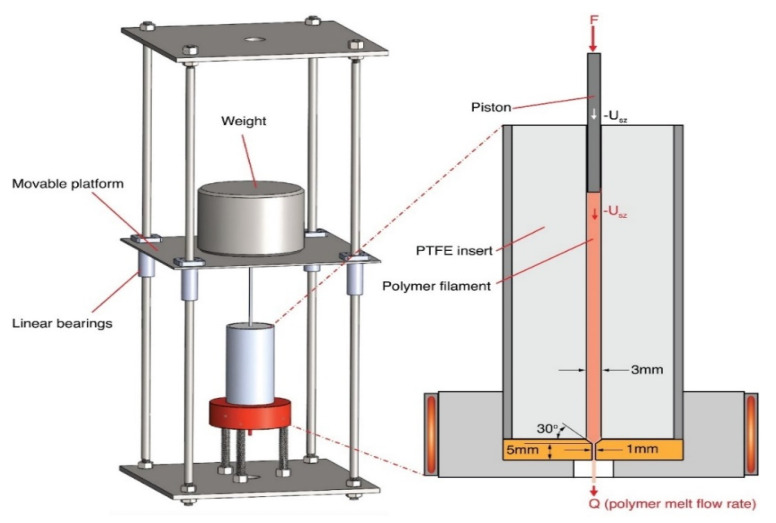
Experimental setup used for validation of the analytical model. Reprinted with permission from reference [32]. Copyright 2018, Elsevier.

In another study, Bellini et al. [59] studied the response of the extrusion system that was analyzed to design a control system for controlling the material flow. A dynamic system model was developed from a derived analytical model to study the system response based on a defined input. The results from this model were compared with experimental data for ABS material. Steady-state error in the dynamic model was observed due to slippage between filament and extruder rollers. In addition, the limitation in the motor torque and power and temperature variation in the liquefier was identified as the reason for the time-delay in response. Likewise, the most appropriate operating temperatures and shear rates for MAFD were explored by Ortega et al. [50]. The analyses were performed on acrylonitrile butadiene styrene (ABS), polylactic acid (PLA), polycaprolactone (PCL), and poly (vinyl alcohol) (PVA). A MAFD nozzle (300 μm) was used to obtain the viscosity models used in the simulation of materials under different operating conditions. The simulation results agreed well with the experimental data. Osswald et al. [32] proposed an analytical model for material melting inside the nozzle, where the applied force governed the maximum melting rate. The model included effects of initial filament temperatures, heater temperature, applied force, nozzle tip angle, capillary diameter, length, and rheological and thermal properties. Experiments performed on ABS material were used to validate the analytical model (Figure 2), and it was concluded that the model accurately predicts melting behavior for forces up to 40 N.

Other than PCL and ABS, Polylactic acid (PLA) is also a widely explored polymer in materials science due to its sustainability and biodegradability [60]. PLA is also an FDA-approved biocompatible material used in medical and food packaging applications [61,62]. Stewart et al. [45] numerically and experimentally investigated the melt flow behavior of the PLA material in the FFF process. The power output of the heating element and temperature profiles within the liquefier were recorded experimentally. The material properties and initial conditions calculated from experimental data were used for 3D modeling of the fluid flow using an accurate extruder geometry. The simulation results concluded that external convective and radiative heat losses play a significant role in material flow realistically.

### 3.2. Fiber Orientation in Polymer Composites

Numerical studies on fused filament fabrication of fiber-reinforced materials are limited. Fiber orientation during the FFF process and mechanical properties are strongly affected by nozzle shape, fiber concentration, material flow rate, and pressure difference. The use of computational modeling software, such as ANSYS^®^, COMSOL^®^, and Moldflow^®^, is reported in the literature. Mostafa et al. [30] investigated the melt flow behavior of iron particle-reinforced Acrylonitrile butadiene styrene (ABS) composites. Two- and three-dimensional numerical simulations were performed using computational fluid dynamics (CFD) software to analyze the temperature, pressure, and velocity changes. Both techniques provided a good correlation in predicting the melt flow behavior. Finally, ABS-iron particle composites were produced and processed to fabricate the samples. Papon et al. [46] studied the effect of FFF process parameters on the melt flow behavior of carbon nanofiber (CNF)-reinforced PLA nanocomposites. A numerical model for non-Newtonian flow was developed to investigate the effect of material properties and different nozzle geometries. Experiments were performed to identify the material rheological properties, and a 3D model of the FFF extrusion channel was developed for simulation. Temperature, pressure, and velocity profiles were obtained, compared with the existing literature, and provided sound agreement.

Fiber orientation in short or continuous fiber-reinforced composites is critical to achieving desired mechanical, thermal, or electrical properties in 3D printed materials. Several experimental studies provided insight into fiber orientation and fiber flow behavior within the nozzle and deposition of extruded beads [63,64,65,66]. However, reported work related to numerical studies will be focused here; readers are referred to the mentioned literature for more details on experimental studies. Kim et al. [52] synthesized silver nanowire (AgNWs)-reinforced photopolymer composites and investigated the effect of nozzle geometry on nanoparticles orientation. Nanocomposites revealed higher dielectric permittivity when 3D printed using a circular nozzle than the flat nozzle, owing to aligned nanowires in the printing direction. Different velocity profiles at nozzle exits obtained from numerical simulations also evidenced the orientation of the nanowires along with the fluid flow for a circular nozzle.

**Figure 3:**
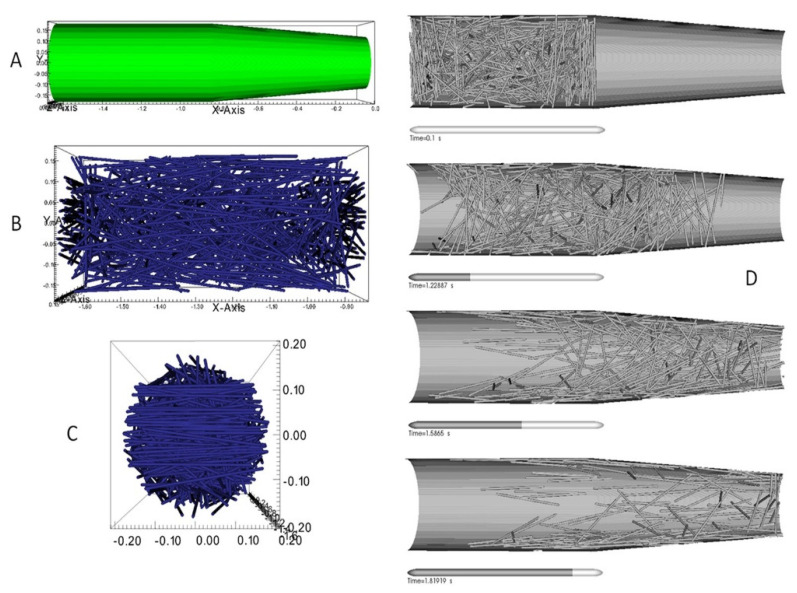
(**A**) Presents the simulations domain considered for analysis. (**B**,**C**) Shows the side views of the extrusion head with random fiber orientations. (**D**) Snapshots of the simulation presenting the fiber flow. Reprinted with permission from reference [53]. Copyright 2017, Science Direct.

Heller et al. [39] studied the effect of nozzle geometry and extrudate swell on fiber orientation of carbon fiber (CF)-reinforced ABS composites. The numerical model was developed in COMSOL integrated with a MATLAB interface. An incompressible fluid flow was modeled considering a Newtonian fluid melt. Fiber orientation was explained using Floger-Tucker [35] and Advani and Tucker [36] models for orientation and isotropic rotary diffusion, respectively. The obtained results were comparable to previously reported studies [37,38]. The authors also performed a parametric analysis to optimize the nozzle geometry for the best achievable modulus in the printing direction. Lewicki et al. [53] performed numerical simulation for CF-reinforced epoxy composites to investigate the melt flow behavior and fiber orientation during the extrusion process. The fibers’ interactions with other fibers, epoxy, and wall were also considered. Randomly oriented fibers were considered at the start, and fiber alignment during the flow was modeled, as shown in Figure 3. The wall-dominated shear alignment was observed from simulation results, resulting in higher fiber orientation along with the flow near to the wall.

**Figure 4:**
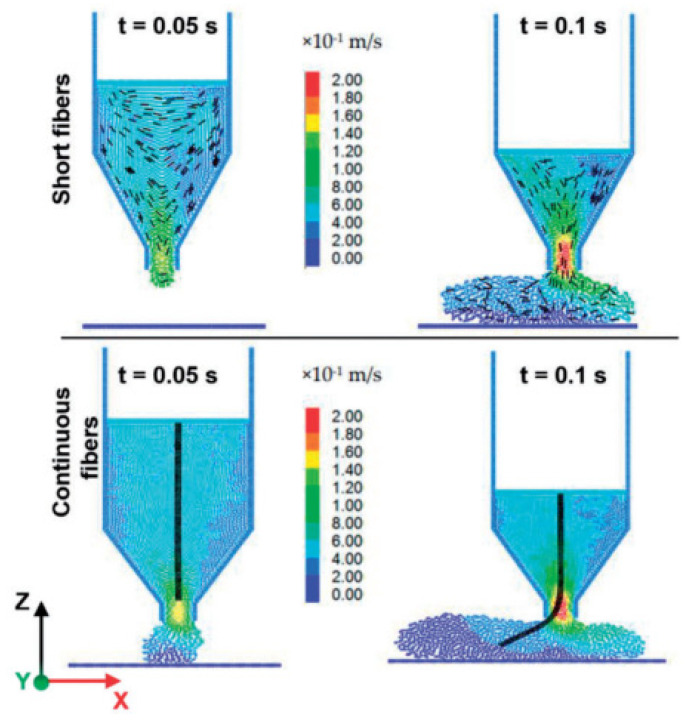
Velocity profiles for short and continuous fiber-reinforced composites. Reprinted with permission from reference [40], Copyright 2017, MDPI.

In addition to the numerical simulations of fiber-reinforced material flow within the nozzle, several studies have reported the fiber orientation within the deposited beads. Bertevas et al. [67] were the first ones to model fiber orientation of 3D printed beads. A Smoothed Particle Hydrodynamics (SPH) framework was employed using a microstructure-based model. The fiber orientation prediction near the nozzle complemented the results from Lewicki et al. [53]. However, it was concluded that fiber orientation near the nozzle should not be approximated as the expected fiber orientation of deposited beads due to significant variations in orientations during deposition. The 3DP process parameters were also identified as critical in fiber orientation within the beads. Yang et al. [40] reported an SPH and discrete element method (DEM) approach to model fiber orientation of short and continuous fiber-reinforced composites. The fiber orientation simulation results for discontinuous fiber composites agreed well with Bertevas et al. [67] observations. The short fibers aligned with the flow direction over time and were supported by the literature, whereas continuous fibers experienced high bending deformations and contact with the nozzle (Figure 4).

### 3.3. Solidification Behavior

**Figure 5:**
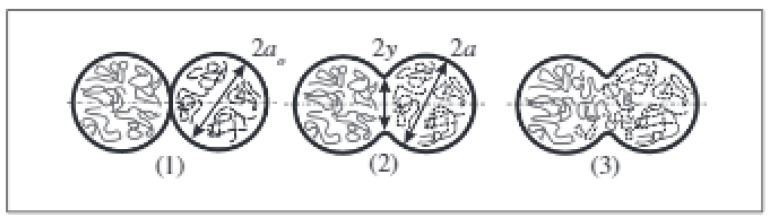
Bead–bead interaction and bond formation mechanism. (**1**) Contact between deposited beads. (**2**) Neck formation. (**3**) Polymer chains diffusion. Reprinted with permission from reference [44]. Copyright 2004, Elsevier.

**Figure 6:**
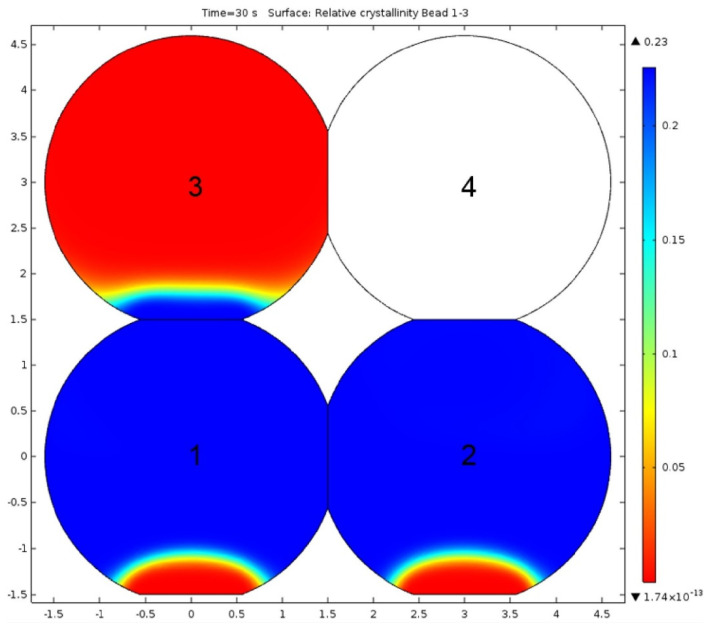
Modeled crystallinity distribution during a print simulation of a simple 2 by 2 bead cross section. Beads 1–3 are active (deposited), while bead 4 is still inactive. Reproduced with permission from reference [20]. Copyright 2018, Elsevier.

The solidification behavior of material governs the bonding between deposited beads, polymer crystallization, and ultimately the mechanical properties of the 3D printed part. When two adjacent beads are deposited, they contact and form necking at the interface [68] (Figure 5). The resulting mechanical properties of 3D printed parts depend upon process parameters and type of polymer. In this section, the numerical and analytical studies related to solidification behavior are presented. Yardimci et al. [69] were the first to model the deposited beads bonding and their thermal interaction with surroundings. A one-dimensional heat transfer model was used, considering beads as grid blocks. Beads surfaces were modeled under convective conditions. Peclet and Biot’s numbers were identified as significant parameters for thermal distributions. Brenken et al. [41] reported a 2D model for CF-reinforced PPS composites’ thermal history and crystallization behavior. A non-isothermal dual crystallization kinetics model was employed to predict crystallization during the solidification process, where beads were individually activated. This model did not consider the thermal variations of the beads in the printing direction. Figure 6 shows the crystallinity distribution of two adjacently deposited beads.

Zhou et al. [42] considered a rectangular cross-section of deposited beads and developed a 3D model to investigate the thermal behavior of ABS material using ANSYS©. A similar methodology of step-wise activation, reported by [41], was used for modeling. The thermal properties of the material were found to have a significant effect on the solidification process. In another study, both convective and radiative heat transfer phenomena were considered to develop a 3D analytical model [43]. The numerical model results found sound agreement with experimental.

**Figure 7:**
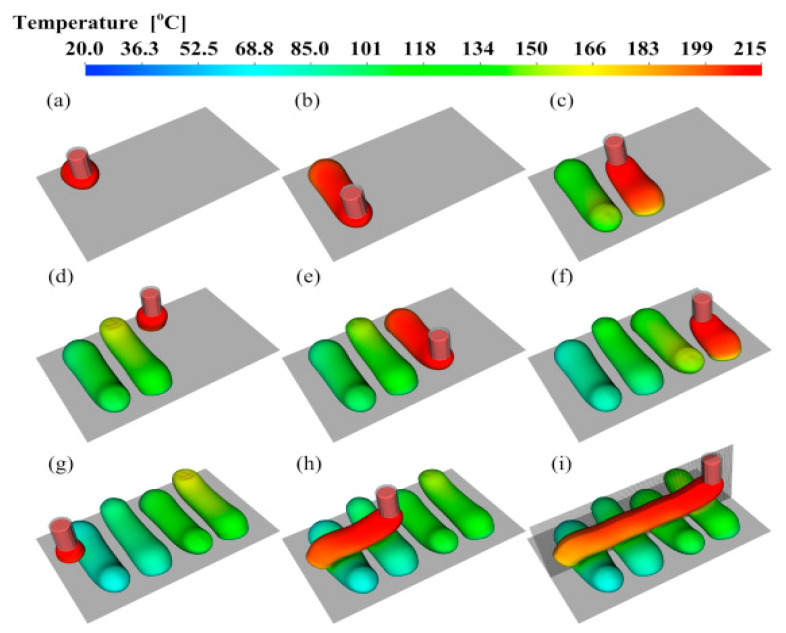
Temperature profiles for simulated printed beads in multi-layer deposition. (**a**–**i**) frames in time order, from the simulation of printing three filaments in parallel. Reprinted with permission from reference [49]. Copyright 2019, ESLEVEIR.

Xia et al. [49] recently presented a bead deposition and solidification model for viscoelastic materials, similar to Liu et al. [70]. The FFF process was modeled using the front-tracking/finite volume method. Three extruded filaments built vertically were simulated considering viscoelastic stresses, and the model was also employed to larger objects (Figure 7). Bellehumeur et al. [44] were the first to predict the bond formation mechanism in 3D printed ABS analytically. A 1D lumped heat transfer model was used to predict the thermal variations. The following governing ordinary differential equation was used:

(1) $$\rho CAv\frac{\partial T}{\partial x}=A\frac{\partial \left(k\frac{\partial T}{\partial x}\right)}{\partial x}-hP\left(T-T_{\infty }\right)$$

The above equation was solved analytically with defined boundary conditions to obtain the following solution for temperature variations during the cooling process:

(2) $$T=T_{\infty }+(T_{0}-T_{\infty })e^{-mx}$$

Subsequently, sintering experiments were performed to investigate the dynamics involved in the bond formation of extruded filaments. The developed model also considered the effect of different printing parameters. It was suggested that better control of the cooling process could assist in controlling the mechanical properties of FFF parts. Further investigation was conducted in another study [71], where a non-isothermal model was used to predict the bonding phenomena. This model was highly sensitive to time and temperature variations. Sun et al. [72] experimentally and numerically investigated the mechanism involved in controlling the bond formation. Sintering temperature had a significant effect on the bond formation and, ultimately, the strength. These observations were also complemented in another work [73]. Costa et al. [74,75] reported a model to consider transient heat transfer during material deposition. Bead–bead, bead–platform, and bead–environment contacts were considered in this model. A MATLAB© code was developed to predict the thermal response and beads adhesion from deposition to complete solidification. Mcllroy et al. [76,77] thoroughly investigated the polymer chains’ effect on crystallization behavior. The models included Rolie-Poly formulas to account for polymer chains diffusion and the impact of shear rate on chains diffusivity, degree of crystallization, and bead–bead chains diffusion.

### 3.4. Residual Stresses and Warpage

The strength and dimensional stability of 3D printed parts are affected by induced residual stresses and warpage. Wang et al. [33] developed a simple analytical model after rigorous simplifications to predict the warp deformation after the FFF process in ABS and quantitatively analyzed all the influencing factors. The following expression was derived to predict the inter-layer warpage (δ):

(3) $$\delta =R\left(1-cos\frac{L}{2R}\right)=\frac{n^{3}\Delta h}{6\alpha \left(T_{g}-T_{e}\right)\left(n-1\right)}*\left\{1-cos\left[\frac{3\alpha L}{n\Delta h}\left(T_{g}-T_{e}\right)\frac{n-1}{n^{2}}\right]\right\}$$

where *R* corresponds to the radius of curvature, *L* represents the section length of the part, *n* presents the number of deposited layers, Δh corresponds to single-layer thickness, and $T_{g}$ and $T_{e}$ represent glass-transition and chamber temperatures. Based on the model analysis, some recommendations were made to reduce the warpage phenomena effectively. Likewise, Armillotta et al. [34] 3D printed ABS samples with varying process parameters and observed wrap deformation for each case. Statistical tools were employed to identify the optimum parameters. The deflection in the 3D printed specimens due to warpage ($\delta_{T}$) was calculated using the following expression:

(4) $$\delta_{T}=\frac{3}{4}\alpha \left(T_{g}-T_{c}\right)\frac{l^{2}h_{0}}{h^{2}}\left(1-\frac{h_{0}}{h}\right)=\frac{3}{4}\alpha \left(T_{g}-T_{c}\right)\frac{l^{2}m\Delta h}{h^{2}}\left(1-\frac{m\Delta h}{h}\right)$$

An analytical model was also derived for both the elastic and plastic behavior of multiple-layer deformations based on the experimental observations. Layer thickness significantly affected the residual stresses, warpage, and surface characteristics.

Xinhua et al. [47] developed a mathematical model for the warpage mechanism in a thin plate of PLA material based on the elastic theory of thin plates. A special part was 3D printed and analyzed by statistical methods to validate the analytical model. Statistical methods were found beneficial to optimize the process parameters, and the proposed model also provided efficient results. However, this model was also based on assumptions, as beads were assumed to be deposited at once, and thermal stresses were neglected. Wijnen et al. [48] replaced the temperature step-function in the model presented by Wang et al. [33] with a physics-based temperature gradient. The proposed model was successfully applied to predict the warpage in thin walls of PLA; however, the limitation of this model was predicting the magnitude of the warpage.

Watanable [51], using a similar 2D model proposed by Bellini [54], performed an extensive study on polypropylene to predict temperature profiles, deposited bead shapes, residual stresses, and warpage deformations. The study was extended to investigate the warpage in PP, and it was suggested to implement PP composites for warpage reduction. The proposed model can be used to simulate novel materials for exploration and applications in the FFF process.

Fitzharris et al. [28] extended the work reported by Watanable [51] to investigate the warpage of high-performance polymer, PPS. Part warpage deformations were obtained experimentally compared to PP results (from Watanable [51]). Material parameters for PPS (including coefficient of thermal expansion CTE, thermal conductivity, heat capacity, and young’s modulus) were individually adjusted to PP values to understand their effect on part warpage. CTE was regarded as the most significant parameter governing the part warpage, residual stresses, and shrinkage from simulation results. In addition, simulation was performed considering material parameters for aluminum nitride (AIN)-filler-reinforced PPS. The incorporation of fillers can alter the CTE, increase thermal conductivity and young’s modulus of PPS. The simulation results for PPS/AIN composite resulted in reduced part warpage.

Zhang and Chou [78] presented a 3-dimensional FEA model for distortion analysis in FFF printed parts. Radiation and conduction heat transfer phenomena were considered in the model, and residual stresses were analyzed using ANSYS©. However, the model lacked the beads interaction during solidification. The sensitivity of residual stresses on process parameters was also studied, and printing speed was identified as an essential parameter in residual stresses. Xia et al. [79] built a more realistic FFF simulation upon their initial work on melt flow [80]. The model was also able to predict the part deformations and material shrinkage over time. The high thermal variations can result in residual stresses; however, this model did not consider the printing bed temperature. In addition to these studies, the same authors also analyzed the shapes of the deposited beads [80].

**Figure 8:**
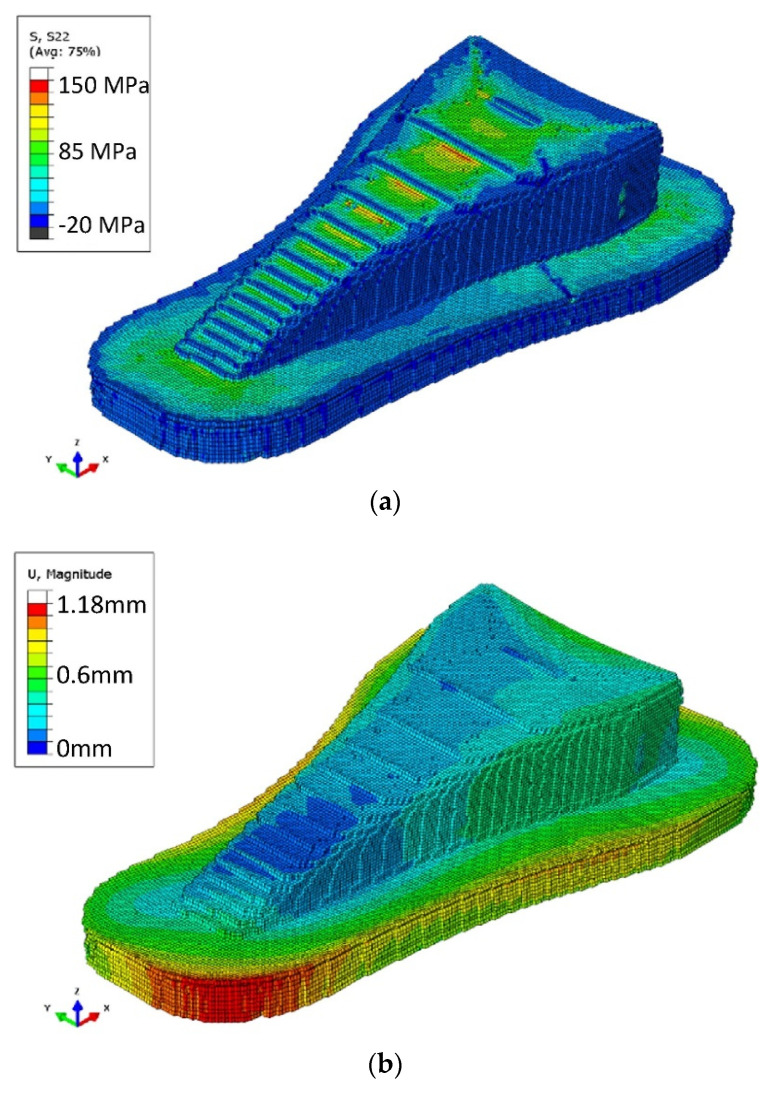
(**a**) Stresses in 3D printed polymer composite part after cooling down (**b**) warpage (exaggerated at a scale of 10). Reprinted with permission from reference [20]. Copyright 2018, Elsevier.

Cattenone et al. [81] developed a framework for 3D simulations on 3D printed parts deformation using ABAQUS©. The mechanical properties were analyzed by varying time-step and mesh, and the results agreed with experimental observations. But this model did not account for bead–bead and bead–print bed interactions. Favaloro et al. [82] were the first to present residual stresses and warpage analysis on polymer composites. ABAQUS© was used to simulate PPS/CF composites using the progressive element activation reported in another work [41]. The model was able to predict the deformations due to crystallization, residual stresses, and part removal. Figure 8 presents the simulation results of residual stresses induced in the 3D printed part after cooling.

Talagani et al. [83] explored the potential use of numerical tools to simulate a full-scale car model. The main aim of this research was to predict the stress concentration areas. The model was able to predict the part deformations due to thermal and residual stresses.

## 4. Future Outlook

The fused filament fabrication process has been under continuous development since the commercial availability of this technology. Several studies reported experimental analysis and virtual modeling of different phases involved in the process. The numerical modeling of material flow inside/outside the printing head and behavior after deposition with promising outcomes have been reported. Based on the extensive literature review performed on numerical simulation techniques, the following research challenges and gaps are identified:

- **Fiber Orientation:** The fiber orientation in deposited beads depends upon the material flow through the nozzle and deposition process. Most of the literature reports the use of Newtonian isotropic fluid properties; however, these materials should be modeled under anisotropic viscous flow conditions. Current modeling software cannot solve fourth or higher-order orientation tensors and cannot consider anisotropic flow characteristics (which is the case with fiber-reinforced composites). Therefore, there is a need for better numerical simulation tools to consider realistic fiber orientation during material flow.
- **Beads Deposition:** Several heat transfer models have been reported in the literature to predict the cooling process of the deposited beads. However, due to the anisotropy involved in the 3DP process, interlayer conduction phenomena need to be considered as thermal conductivities of deposited beads change with the fiber orientation.
- **Interface and Bonding:** Bonding between the subsequent layers is highly correlated with the interface; therefore, the presence of fibers on the bead surface can affect this process. In addition, the necking phenomenon is derived by the gradients of surface tension is also influenced by the bead surface morphology. Finally, the material behavior (crystalline or amorphous) will reflect its viscosity, which ultimately affects the bonding process; therefore, it must be accounted for accurate process modeling.
- **Integrated Simulation Models:** The FFF process is a complex multi-stage process as described in this paper. However, most reported computational work either focused on the material flow inside the liquefier or material behavior after deposition and is not as mature as the experimental literature. Therefore, there is a need for integrated studies considering all these phases of the FFF process (i.e., melt flow behavior inside/outside the nozzle, material deposition, solidification behavior, bond formation, and warpage and residual stresses).
- **Model Validation:** The validation of numerical and analytical models is vital through experimental studies. Limited studies compared the numerical simulation results with experimental work, which is essential for validating and broader application of these models.
- **Materials Portfolio:** Materials portfolio for the FFF process is rapidly growing. However, few materials (such as PLA and ABS) are considered for numerical and analytical modeling of process or material behavior. The researchers should focus on implementing existing models to a broader range of materials or develop models for materials not yet considered in the literature.
- **Polymer Composites:** Two-phase materials (composites) are also barely considered for the numerical modeling of material or the FFF process. The most reported models address linear amorphous polymers. Different polymers exhibit different characteristics, such as bare PLA and ABS act as amorphous materials; however, PBT, PA12, and PEEK exhibit a semi-crystalline nature [84,85,86]. Moreover, the addition of the reinforcing phase can alter the nature of the resulting composite material, e.g., PLA acts as semi-crystalline material with tricalcium phosphate (TCP) [87]. The effect of reinforcement type and process parameters on polymer nature (amorphous or crystalline) will be worth addressing.

## 5. Conclusions

This study provides a comprehensive review of state-of-the-art scientific achievements in numerical simulations for the FFF process of polymers and their composites. The first section presents an in-depth discussion on the physical phenomena involved in the FFF process and highlights the multi-level complexity. The subsequent section discusses the research efforts, specifically on numerical simulation techniques reported in the literature for the FFF process.

The future of 3DP processes, specifically the FFF process, is promising due to research and development interest. However, several challenges are still faced. The focused research on the gaps mentioned above could further improve the process and material design.

Currently, the FFF process is being explored extensively; however, the numerical simulation approaches are still empirically calibrated. This work has identified several issues persisting the wide use of numerical simulation techniques for its development. Addressing these research challenges will enable a more realistic and reliable prediction of the FFF process.

## Figures and Tables

**Table 1:** Summary of studies reported on the numerical modeling of fused filament fabrication process.

Material	Additives	Analysis	Tools	Highlights	Ref.
PCL	-	Melt Flow Behavior	ANSYS©	Observation of velocity, pressure, and thermal variations. Filament velocity at the inlet of the channel was varied. Variation in nozzle shape and the angle at the exit. Material liquified within 35% of the channel length.	[29]
ABS	Iron Particles (10%)	Melt Flow Behavior	ANSYS©	Study of velocity, temperature, and pressure variations. Fabrication and characterization of composites. Promising simulation results for melt flow behavior and process optimization.	[30]
ABS	-	Swelling and Filament Cooling	Dieplast© and EFD Lab	Potential for using fine nozzle diameters for MAFD. Nozzle temperature regarded as primary contributor to die swelling. Temperature variations along the nozzle length. Volume of flow 215 times lower than conventional nozzles.	[31]
ABS	-	Melting Inside Nozzle	Mathematical Model	Analytical model for melting inside the nozzle. Material flow was controlled by applied force. Experimental validation of proposed model. Good prediction of material behavior for force up to 40 N.	[32]
ABS	-	Warpage	Mathematical Model	Simple model for warpage deformation was developed. All influencing parameters (layer number, chamber temperature, material shrinkage rate) were quantitatively analyzed. Recommendations to avoid warp deformation.	[33]
ABS	-	Warpage	Mathematical Model	Analytical model based on experimental observations was developed. Model can predict multi-layer deformation of 3D printed parts. Strong effect of layer thickness on warpage was observed.	[34]
ABS	CF	Fiber Orientation	COMSOL© MATLAB©	Effect of nozzle geometry and extrudate swell. Used Floger-Tucker [35] and Advani and Tucker [36] models. Comparable results to previously reported studies [37,38].	[39]
ABS	CF	Fiber Orientation	SPH-DEM	Both short and continuous fiber composites. Highly aligned short fibers with material flow over time. Lower printing speeds recommended for continuous fiber composites to avoid nozzle wear and fiber breakage.	[40]
ABS	-	Solidification	ANSYS©	Rectangular cross-section of deposited beads. 3D model to investigate thermal behavior. Similar stepwise activation, as reported by [41]. Thermal properties of the material were found to have a significant effect on the solidification process.	[42]
ABS	-	Solidification	Mathematical Model	Both convective and radiative heat transfer phenomena were considered to develop a 3D model. The numerical model results found sound agreement with experimental results.	[43]
ABS	-	Bond Formation	Mathematical Model	First model to predict the bond formation mechanism. 1D lumped heat transfer model was used. The model also considered the effect of printing parameters. Concluded better control of the cooling process to control mechanical properties of FFF parts.	[44]
PLA	-	Melt Flow Behavior	ANSYS©	Experimentally obtained liquefier temperature profile and heating element power output. Detailed 3D model with all assemblies. External heat transfer mechanisms were found more significant.	[45]
PLA	CNF (0–1%)	Melt Flow Behavior	ANSYS©	Rheological and mechanical properties obtained experimentally. Simulation of non-Newtonian fluid flow using 3D model. Results agreed well with existing numerical models.	[46]
PLA	-	Warpage	Mathematical Model Statistical Analysis	2D analytical model based on theory of thin plates. Taguchi’s method was used for design of experiments. ANOVA and S/N ratio were used to optimize the process parameters. Proposed model was found efficient but thermal stresses were ignored.	[47]
PLA	-	Warpage	Mathematical Model	Successful prediction of distortion for PLA thin walls. Limitation in terms of warpage magnitude.	[48]
PLA	-	Bead Deposition and Solidification	Mathematical Model	A model for viscoelastic materials The front-tracking/finite volume method was used. Three extruded filaments built vertically were simulated considering viscoelastic stresses. The model was also employed to larger objects.	[49]
ABS, PCL, PLA	-	Swelling and Process Conditions	SolidWorks©	Nozzle equipped with pressure and temperature sensors. High shear rates resulted in a higher swell. Viscosity models were obtained from experimental analysis. Simulations agreed well with experimental data.	[50]
PP	-	Melt Flow Bead Shape Residual Stresses Warpage	ANSYS©	Experimental and numerical investigation. Special focus on warpage and mechanical properties. Good agreement of numerical simulation results with experimental observations.	[51]
PPS	AIN	Warpage	ANSYS©	Extended work from Watanable [51]. Analysis of most significant material parameter. CTE concluded most significant for part warpage. Composite materials with lower CTE can reduce warpage.	[28]
PPS	CF	Solidification Crystallization	COMSOL©	2D model for thermal history and crystallization behavior. Used non-isothermal dual crystallization kinetics model. Individual activation of beads. Thermal variations of the beads in the printing direction were not considered.	[41]
Photo Polymer	AgNWs (1.6 vol%)	Nanofiller orientation	ANSYS©	Nozzle geometry effect on fiber orientation. Aligned nanowires for circular nozzle. Different velocity profiles at nozzle exits.	[52]
Epoxy	CF (8 vol%)	Fiber Orientation	STARCCM+	Melt flow within the nozzle. Fibers interactions with other fibers, epoxy, and wall. Higher fiber orientation near to the wall.	[53]

## Data Availability

Data sharing not applicable.
